# The Utility of Major Salivary Gland Ultrasonographic Parameters in the Diagnosis of Sjögren Syndrome

**DOI:** 10.1155/2019/1716848

**Published:** 2019-12-14

**Authors:** Alexandru Caraba, Flavia Corina Babalic, Stela Iurciuc, Mircea Iurciuc

**Affiliations:** ^1^Department of Internal Medicine, Division of Rheumatology, “Victor Babeș” University of Medicine and Pharmacy, Timișoara, Romania; ^2^Department of Internal Medicine, City Hospital, Timișoara, Romania; ^3^Department of Cardiology, “Victor Babeș” University of Medicine and Pharmacy, Timișoara, Romania

## Abstract

**Objective:**

To investigate ultrasonographically the salivary glands and to correlate ultrasonographic parameters with focus score, serum beta-2-microglobulin, and stimulated salivary flow rate.

**Material and Methods:**

32 patients with primary Sjögren's syndrome (pSS) and 32 healthy controls were enrolled in this case-control study, performed in the Department of Internal Medicine, Division of Rheumatology, “Victor Babeș” University of Medicine and Pharmacy, Timișoara, Romania. All the patients and controls were examined by salivary gland ultrasonography (B-mode, color and spectral Doppler, and sonoelastography), determining the following parameters: salivary gland ultrasonography (SGUS) score, resistive index (RI) of transverse facial artery, and shear wave velocity (SWV). Serum beta-2-microglobulin and stimulated saliva amount were determined in all the patients and controls. Minor salivary gland biopsy with focus score assessment was done in pSS patients.

**Results:**

Patients with pSS presented higher SGUS score and parotid and submandibular SWV and reduced RI of transverse facial artery than controls (*p* < 0.0001). In pSS patients, statistically significant correlations were identified between assessed ultrasonographic parameters and focus score, serum beta-2-microglobulin, and respective stimulated saliva flow (*p* < 0.0001).

**Conclusions:**

This study highlighted statistically significant correlations between salivary gland ultrasonographic parameters and focus score, serum beta-2-microglobulin, and stimulated saliva flow.

## 1. Introduction

Primary Sjögren's syndrome (pSS), a chronic autoimmune disorder, is characterized by lymphocytic infiltration and then destruction of the exocrine glands, especially the salivary and lachrymal glands. The main symptoms of pSS are represented by dry mouth and eyes. But in addition to glandular involvement, pSS may have systemic manifestations, some of them being very serious, especially lymphoma development. Therefore, an early diagnosis and an appropriate therapy are very important goals for these patients [1].

Over time, several classification criteria for pSS have been developed. The new classification criteria developed in 2016 by the American College of Rheumatology (ACR)/European League Against Rheumatism (EULAR) included minor salivary gland biopsy (MSGB) [2]. This criterion is required in cases with negative anti-SSA/SSB antibodies. But MSGB is an invasive procedure and on the other hand is dependent on the pathologist's experience [1]. Salivary and lacrimal glands are affected by an intense lymphocytic and plasma cell infiltration and then destruction of these glands. CD4+ T lymphocytes and B-lymphocytes represent approximately 90% of the infiltrating cells found in the inflammatory glandular infiltrate. Along with them, plasma cells, CD8+ T lymphocytes, T regulatory cells, CD56+ natural killer cells, macrophages, and myeloid and plasmacytoid dendritic cells are identified, too. B-lymphocytes are most commonly identified in inflammatory infiltrate as the severity of the pSS increases [3]. These histopathological aspects have ultrasonographic correspondence through the inhomogeneous structure of the glands with scattered multiple small, oval, hypoanechoic or hyperechoic areas, usually well defined (due to multiple cysts or calcifications, respectively); irregularity of the margins; presence of peri-intraglandular lymph nodes; and increased parenchymal blood flow [4].

Over the last 10 years, the interest in the use of ultrasonography in the diagnosis of pSS has greatly increased. Many studies have shown the importance of ultrasonography in the assessment of salivary glands in pSS patients. By salivary gland ultrasonography (SGUS), a noninvasive, repeatable method, the structural changes and abnormalities in vascularization of salivary glands are documented in pSS patients. This method allows the monitoring of glandular abnormalities throughout the pSS evolution. On B-mode ultrasonography, several scoring systems were developed in order to characterize structural abnormalities in pSS patients (De Vita et al., Hocevar et al., Cornec et al., Takagi et al.). The main parameters evaluated within these scores are parenchymal echogenicity and homogeneity, the presence of hypoechoic areas and hyperechoic foci, and visibility of glandular borders. Abnormalities of salivary gland vascularization are also evidenced by color and spectral Doppler ultrasonography of transverse facial artery, which demonstrates resistive index (RI) reduction in pSS [5–10].

It should be specified that homogeneous glandular parenchyma or mild abnormalities of salivary glands do not exclude pSS. Sonoelastography represents a novel ultrasonographic method which evaluates the tissue stiffness. Acoustic radiation force impulse (ARFI) imaging is a novel type of sonoelastography that allows the assessment of tissue stiffness by evaluating wave propagation. ARFI imaging with Virtual Touch Quantification (VTQ) represents a sonoelastography method that provides an objective numerical assessment of tissue stiffness [11]. Tissue stiffness is quantified by the speed of the shear waves, as shear wave velocity (SWV), expressed as meters per second (m/s). Stiffer tissues are associated with a higher SWV [12]. Tissue stiffness has been shown to correlate with the degree of fibrosis and inflammation. Mononuclear inflammatory infiltrate and fibrosis characterize pSS histopathologically. Thus, the patients with pSS show increased values of parotid and submandibular gland stiffness, which may be useful for diagnosis of this syndrome [13]. In their preliminary study, Chen et al. reported that by combining the salivary gland ultrasonographic score and SWV values, the diagnosis of pSS was improved [14].

The pSS activity is related to the B-cell hyperactivity, reflected by high levels of serum beta-2-microglobulin [15]. The chewing gum test and Saxon test are used in order to appreciate the amount of stimulated saliva, the Saxon test being considered just as good as or even better than the chewing gum test [16].

The aim of this study was to assess the diagnostic value of salivary gland ultrasonography (B-mode, Doppler, and ARFI imaging with VTQ) and to establish the correlations between ultrasonographic parameters and focus score and on the other hand with serum beta-2-microglobulin.

## 2. Material and Methods

### 2.1. Patients

This case-control study was performed in the Department of Internal Medicine, Division of Rheumatology, Timișoara, Romania, between July 2016 and August 2019 on a group of 32 patients with primary Sjögren's syndrome and 32 healthy subjects, matched for age and gender, as controls. All patients fulfilled the 2016 ACR/EULAR Classification Criteria for primary Sjögren's syndrome [2]. Exclusion criteria were represented by age under 18 years, overlap syndromes, secondary Sjögren's syndrome, sicca symptoms related to hepatitis C infection, acquired immunodeficiency syndrome, sarcoidosis, IgG4-related disease, previous head and neck radiation therapy, graft versus host disease, diabetes mellitus, amyloidosis, chronic kidney disease, pregnancy or breastfeeding women, current use of drugs that might decrease salivary gland function, and current smokers. The controls were enrolled from healthy attendants coming to the Rheumatology Division with the pSS patients. All the patients and controls gave their informed consent. The study was approved by the Ethics Committee of “Victor Babeș” University of Medicine and Pharmacy, Timişoara, Romania. This study respects the Declaration of Helsinki.

### 2.2. Methods

Antinuclear antibodies, anti-SSA, and anti-SSB antibodies were determined using indirect immunofluorescence (HELMED). The rheumatoid factor was determined by the latex agglutination test.

Serum beta 2-microglobulin was performed using an immunoenzymometric assay with chemiluminescence detection (CLIA-serum).

For focus score assessment, the minor salivary glands are very accessible for calculating the focus score. They are placed under the inner surface of the lip. After numbing with Lidocain 10%, the surgeon made an incision on the inner surface of the lip and then removed 5-7 glands using sterile tweezers. Using a hematoxylin-eosin stain, the pathologist identified tight clumps of lymphocytes (≥50), called foci. Their density on a surface of 4 mm^2^ defined the focus score [17].

The Saxon test is used in order to measure the amount of stimulated saliva. A dry gauze sponge folded twice at 90° angles, having the final dimension of 5 × 5 cm, was weighed. Then, the patient was invited to chew the gauze sponge for 2 minutes, at a chewing rate of 120 times during the measurement period. After that, the gauze sponge was weighed again. The difference between the final and the initial weights represented the amount of stimulated saliva [16].

Ultrasonographic assessment of salivary glands was done using a Siemens ACUSON A2000 device equipped with a multifrequency linear transducer at a frequency of 5-14 MHz.

First, B-mode ultrasonography was performed. The 4 major salivary glands (bilateral parotid and submandibular glands) were assessed in the longitudinal and transverse planes. Thyroid echogenicity was used to compare to the salivary gland echogenicity. For parotid gland examination, the patient was invited to turn his head to the side opposite the side being examined; the parotid gland was scanned in the retromandibular fossa, anterior to the ear and sternocleidomastoid muscle. For submandibular gland examination, the patient was invited to tilt back his head in a supine position.

Hocevar's SGUS score was used (ranged from 0 to 48). The following parameters were recorded: parenchymal echogenicity evaluated in comparison with thyroid parenchyma (graded as 0 or 1), glandular homogeneity (graded from 0 to 3), the presence of hypoechoic areas in the glandular parenchyma (graded from 0 to 3) and hyperechoic foci in parotid glands (graded from 0 to 3) and in submandibular glands (graded from 0 to 1), and visibility of glandular borders (graded from 0 to 3). The SGUS score was calculated by the summation of these parameter grades for all 4 glands [6].

The transverse facial artery was identified by color Doppler ultrasonography, in front of the external auditory canal. Then, by spectral Doppler ultrasonography, the resistive index (RI) was determined [18].

Finally, ARFI (VTQ) was performed. The transducer was gently placed to the face surface with a sufficient amount of ultrasound gel. The parotid glands were evaluated in a longitudinal plane, avoiding interference with bones and salivary ducts. The submandibular glands were evaluated in a longitudinal plane. The most important aspect of this procedure was represented by avoiding the main vascular branches, identified by color Doppler imaging. In order to have a comprehensive evaluation of salivary glands, six VTQ measurements of the SWV were obtained in the central, peripheral, and subcapsular areas [13].

### 2.3. Statistical Analysis

Data are presented as the mean ± standard deviation. Statistical analyses were performed using Student's *t*-test, ANOVA test, and Pearson's correlation. Differences were considered statistically significant at the value of *p* < 0.05.

## 3. Results

Baseline demographic data of pSS patients and controls are presented in Table 1.

Schirmer's test was positive in all the patients, with the mean values of 2.68 ± 1.37 mm. The focus score had the mean values of 3.93 ± 1.21. Antinuclear antibodies, anti-SSA and anti-SSB antibodies, and rheumatoid factor were demonstrated in all patients. The mean values of the rheumatoid factor were 125.66 ± 72.03 IU/dl.

**Figure 1:**
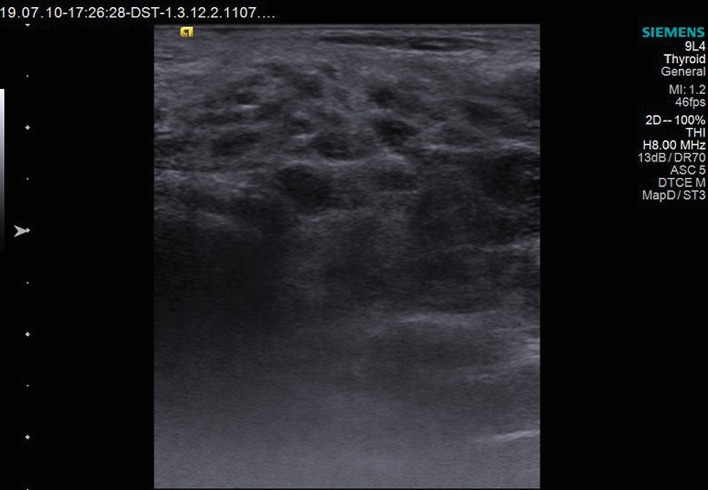
B-mode ultrasonography of the parotid gland.

**Figure 2:**
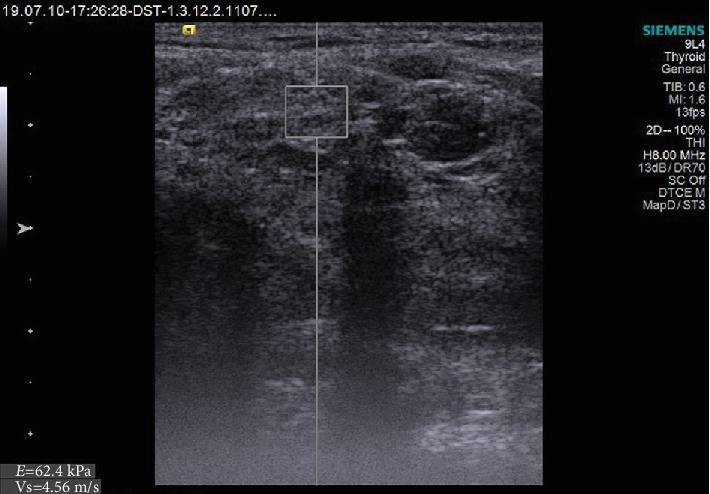
ARFI imaging-VTQ of the parotid gland.

The assessed ultrasonographic parameters showed significant differences in pSS patients *vs.* controls. SGUS score was higher in pSS patients than in controls (*p* < 0.0001). On the other side, RI of the transverse facial artery had lower values in pSS patients than in controls (*p* < 0.0001). SWV reported higher velocities both in parotid (Figures 1 and 2) and submandibular glands in pSS than in controls (*p* < 0.0001). All these differences were statistically significant (Table 2).

All these differences of ultrasonographic parameters between pSS patients and controls reflected the consequence of the glandular histopathological changes in pSS. Because the focus score is the marker of glandular histopathological changes in pSS, the correlations between ultrasonographic parameters and focus score were investigated. They are presented in Table 3.

Statistically significant correlations were observed between the focus score and assessed ultrasonographic parameters. The strongest correlations were identified between the focus score and ARFI imaging with VTQ parameter (SWV).

The methods of ultrasonographic examination (B-mode, color and spectral Doppler, and ARFI (VTQ)) are complementary to one another, and the use of all of them increases the accuracy of the pSS diagnosis. The correlations between ultrasonographic parameters (SGUS score, RI of transverse facial artery, and parotid and submandibular SWV) are presented in Table 4.

**Figure 3:**
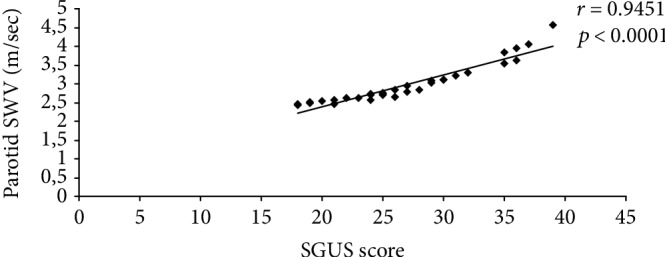
Correlation between SGUS score and parotid SWV.

Statistically significant correlations were observed between ultrasonographic parameters in studied pSS patients (a negative one between SGUS score and RI of transverse facial artery and positive one between SGUS score and parotid SWV and respective submandibular SWV) (Figure 3).

Serum beta-2-microglobulin, a marker of B-cell hyperactivity, presented higher values in pSS than in controls (2.84 ± 0.55 mg/l *vs.*1.86 ± 0.44 mg/l, *p* < 0.0001). Higher values of serum beta-2-microglobulin were correlated with SGUS score (*r* = 0.9396, *p* < 0.0001).

The amount of stimulated saliva was lower in patients than in controls, statistically significant (1.17 ± 0.38 g/2 min vs. 6.33 ± 0.61 g/2 min, *p* < 0.0001).

The correlations between the amount of stimulated saliva, evaluated by the Saxon test, and ultrasonographic parameters are presented in Table 5. Severe histological changes reflected by higher values of SGUS score and parotid and submandibular SWV and reduced values of RI of transverse facial artery were associated with the reduction of the amount of stimulated saliva.

## 4. Discussion

Sjögren's syndrome, a complex autoimmune disease, is characterized by a broad spectrum of clinical and serological manifestations, dry eyes and dry mouth being the most common complaints [19].

Primary Sjögren's syndrome is one of the most common autoimmune disorders, having a prevalence between 0.1% and 4.6%. The incidence and prevalence rates were approximately 6 times higher among women than men [20].

The present study revealed that in pSS patients, ultrasonography (B-mode, color and spectral Doppler of transverse facial artery) and sonoelastography are valuable tools in assessing salivary gland involvement in pSS. Significant correlations between ultrasonographic parameters (SGUS score, RI of transverse facial artery, and parotid and submandibular SWV) and focus score, respectively, and amount of stimulated saliva and serum beta-2-microglobulin were highlighted.

Studying 77 patients with pSS and 79 patients with sicca symptoms without pSS, Salaffi et al. showed statistically significant differences between the values of the mean ultrasonography scores in the two groups of studied subjects (*p* < 0.0001) [21]. Cornec et al. revealed that the structural changes, as well as Doppler waveform analysis of parotid and submandibular glands, may improve the diagnostic performance in pSS patients [7]. Studying 94 patients with pSS and 44 patients with idiopathic sicca syndrome, Lee et al. showed that the pSS patients presented a significantly higher SGUS score than the patients with idiopathic sicca syndrome (24.5 ± 13*vs.*6 ± 3.75, *p* < 0.001). The authors revealed that a SGUS score cut-off of ≥14 had a sensitivity of 80.9% and a specificity of 95.5% for the diagnosis of pSS [1]. Jousse-Joulin et al. compared 16 pSS patients to 9 controls, using B-mode ultrasound features of parotid glands and Doppler waveform analysis of the transverse facial artery, before and after 12 weeks of intravenous rituximab treatment. Compared to controls, before treatment, pSS patients presented abnormalities in the salivary gland structure (*p* < 0.0001) and parotid size (*p* = 0.001). Basal RI values were significantly reduced in the pSS patients compared with the controls (0.75 ± 0.05*vs.*0.81 ± 0.42, *p* < 0.005). Rituximab determined B-lymphocyte infiltrate depletion and decreased the glandular inflammation. After 12 weeks, gland size decreased significantly (*p* = 0.002 for parotid glands and *p* = 0.001 for submandibular glands). RI of transverse facial artery increased, but without statistical significance. These findings offered an indirect proof of the correlation between histopathological and ultrasonographic parameters in pSS patients. Moreover, in the pSS patients, the severity of gland damage assessed by ultrasound correlated with the RI [22]. The studies performed by Chikui et al. and Gritzmann et al. revealed the abnormalities in salivary gland vascularization in these patients [23, 24].

The pSS patients present a glandular hypervascularization with a diffused pattern, directly related to the extent of parenchymal changes. This glandular hyperemia is associated with the inflammation degree. Glandular hypervascularization may result in a reduction in the RI of the transverse facial artery [25].

In recent years, the importance of sonoelastography in pSS has been studied. Knopf et al. identified the higher SWV values in the pSS *vs.* non-pSS patients (2.86 ± 0.07 m/s *vs.*2.15 ± 0.11 m/s, *p* < 0.0001). The authors had shown that ARFI imaging had a diagnostic sensitivity of 81% and specificity of 67%, respectively [26]. Samier-Guérin et al. reported that the ARFI imaging characterized the abnormal architectural changes in the parotid glands (*p* = 0.001) [27]. Zhang et al. evaluated salivary gland stiffness in pSS patients via ARFI imaging, including VTQ. They demonstrated that the parotid gland VTQ values were significantly higher in pSS patients than in controls (*p* < 0.01) [28]. Chen et al. studying submandibular and parotid glands in the early stage of pSS patients, non-pSS patients with sicca symptoms, and healthy controls identified statistically significant differences in SWV among these patients and controls (*p* < 0.01) [13]. In a study published by Turnaoglu et al. using ARFI imaging with VTQ of parotid and submandibular glands, the authors reported that in pSS patients, the mean values of SWV of parotid and submandibular glands were significantly higher in the pSS patients than in the healthy control group (*p* < 0.001). The authors established the cut-off of SWV values of 1.98 m/s for submandibular glands, and 1.93 m/s for parotid glands, and reported that the mean SWV values of parotid glands were higher than those of the submandibular glands (*p* < 0.001) [12]. As in other studies, the present study highlighted different values of SWV in parotid and submandibular glands (2.98 ± 0.53 m/sec *vs.*2.63 ± 0.59 m/sec), related to the different histological composition of the parotid and submandibular glands (lymphocytic infiltrates and glandular fibrosis occur earlier and are more severe in the parotid glands than in the submandibular glands) [12, 26].

In the present study, statistically significant correlations were demonstrated between the focus score and ultrasonographic parameters (SGUS score, RI of transverse facial artery, and parotid and submandibular SWV). Salaffi et al. reported that the ultrasonographic glandular abnormalities were strongly correlated with severity of the inflammatory infiltrate (*p* = 0.01) [29]. El Miedany et al. identified in their study a significant correlation between the salivary gland ultrasound score and the histopathological score (*r* = 0.82, *p* < 0.001) [30]. Using the ultrasonographic scoring system described by De Vita et al., Luciano et al. described a significant correlation between the SGUS score and the minor salivary gland biopsy/focus score (*r* = 0.484, *p* < 0.0001) [31]. Analyzing retrospectively the records of 85 suspected pSS patients, Astorri et al. identified that an overall concordance between the ultrasound and the histology was 91% [32]. Cornec et al. reported that the SGUS score correlated positively with the focus score (*r* = 0.61) [33]. Mossel et al. assessed 103 consecutive outpatients with clinically suspected pSS by glandular ultrasonography (using Hocevar scoring system) and parotid and respective minor salivary gland biopsy. These authors obtained an absolute agreement between SGUS score and parotid (83%) and minor salivary (79%) gland biopsies [34].

B-cell hyperactivity represents a key hallmark of the pSS activity, reflecting in hypergammaglobulinemia, autoantibody production, and systemic manifestations of this disease. Serum beta-2-microglobulin is a biomarker of the lymphocyte hyperactivity [19]. This study revealed that the pSS patients presented higher values of serum beta-2-microglobulin than controls (2.84 ± 0.55 mg/l *vs.*1.86 ± 0.44 mg/l, *p* < 0.0001). Higher values of serum beta-2-microglobulin were correlated with SGUS score (*r* = 0.9396, *p* < 0.0001). The subjects with higher SGUS score had more frequently significant systemic complications and increased disease activity and markers of lymphoma development [25].

High ultrasonographic scores are associated with the reduction of salivary flow. Cornec et al. reported that the SGUS score correlated negatively with the unstimulated salivary flow rate (*r* = ‐0.68, *p* < 0.001) [33]. Hammenfors et al. demonstrated on 94 patients with pSS that the ultrasonographic scores of salivary glands correlated with unstimulated (*r* = ‐0.424, *p* < 0.001) and stimulated saliva (*r* = ‐0.503, *p* < 0.001) [35]. In the study performed by Lee et al., the SGUS score was inversely correlated with the unstimulated salivary flow rate (*r* = ‐0.578, *p* < 0.001) [1].

All of the study pSS patients presented ultrasonographic changes of major salivary glands (SGUS score between 18 and 39, with the mean value of 26.90 ± 5.94). But the absence or mild abnormalities of salivary glands do not exclude pSS [5–10]. In these cases, only the salivary gland biopsy establishes the pSS diagnosis. Therefore, histological and ultrasonographic exams can be considered as two complementary methods, at least in the early stages of pSS evolution.

This study has some limits. First, the relatively small number of pSS patients is one of the limits of this study. Secondly, it was a case-control study and the information regarding the subsequent evolution of the pSS patients according to the ultrasonographic parameters is missing.

## 5. Conclusions

Ultrasonographic assessment of the major salivary gland (B-mode, color and spectral Doppler, and ARFI sonoelastography) revealed strong correlations between ultrasonographic parameters and histological picture, stimulated salivary flow, and serum beta-2-microglobulin.

Histological and ultrasonographic exams can be considered as two complementary methods, at least in the early stages of pSS evolution.

## Figures and Tables

**Table 1:** Demographic data in pSS patients and controls.

Parameter	Value (mean ± standard deviation)	
	pSS patients	Controls
Sex (*n* (%))	32	32
Males	8 (25%)	8 (25%)
Females	24 (75%)	24 (75%)
Mean age (years)	52.28 ± 10.08	51.78 ± 7.46
Mean length of sicca symptom evolution (years)	3.79 ± 1.18	—
The drugs used by the pSS patients in the moment of investigation	Hydroxychloroquine (16 patients)	—
	Azathioprine (10 patients)	
	Methotrexate (6 patients)	
Extraglandular involvement in pSS patients		—
Malignant lymphoma	3 patients (9.37%)	
Articular involvement	8 patients (25%)	
Pulmonary involvement	5 patients (15.62%)	
Renal involvement (renal tubular acidosis type 1)	7 patients (21.87%)	
Cutaneous vasculitis	3 patients (9.37%)	

**Table 2:** Ultrasonographic parameters assessed in pSS patients and controls.

Parameter	pSS patients	Controls	*p*
SGUS score	26.90 ± 5.94	5.62 ± 1.28	<0.0001
RI	0.73 ± 0.01	0.81 ± 0.02	<0.0001
Parotid SWV (m/sec)	2.98 ± 0.53	1.83 ± 0.07	<0.0001
Submandibular SWV (m/sec)	2.63 ± 0.59	1.89 ± 0.04	<0.0001

**Table 3:** Correlations between the focus score and ultrasonographic parameters.

Correlation between focus score and	*r*	*p*
SGUS score	0.6774	<0.0001
RI of transverse facial artery	-0.6318	<0.001
Parotid SWV (m/sec)	0.7129	<0.0001
Submandibular SWV (m/sec)	0.7334	<0.0001

**Table 4:** Correlations between ultrasonographic parameters.

Correlation between SGUS score and	*r*	*p*
RI of transverse facial artery	-0.8423	<0.0001
Parotid SWV (m/sec)	0.9451	<0.0001
Submandibular SWV (m/sec)	0.9205	<0.0001

**Table 5:** Correlations between the Saxon test and ultrasonographic parameters.

Correlation between Saxon test and	*r*	*p*
SGUS score	-0.8325	<0.0001
RI of transverse facial artery	0.6448	<0.0001
Parotid SWV (m/sec)	-0.6643	<0.0001
Submandibular SWV (m/sec)	-0.6593	<0.0001

## Data Availability

All the data processed in this study come from the patient records. Because of ethical concerns (the patient privacy), the access to these data is restricted. We have the permission to make public only the average values and the standard deviations of the analyzed parameters, without presenting the patient records. The patient identification data were deleted from the ultrasonographic images presented in the paper.
